# Quantitative Analysis of Peripheral Tissue Perfusion Using Spatiotemporal Molecular Dynamics

**DOI:** 10.1371/journal.pone.0004275

**Published:** 2009-01-26

**Authors:** Yujung Kang, Myunghwan Choi, Jungsul Lee, Gou Young Koh, Kihwan Kwon, Chulhee Choi

**Affiliations:** 1 Department of Bio and Brain Engineering, KAIST, Daejeon, Korea; 2 Department of Biological Sciences, KAIST, Daejeon, Korea; 3 Graduate School of Medical Science and Engineering, KAIST, Daejeon, Korea; 4 KI for the BioCentury, KAIST, Daejeon, Korea; 5 Department of Cardiology, School of Medicine, Ewha Womans University, Seoul, Korea; University of Arizona, United States of America

## Abstract

**Background:**

Accurate measurement of peripheral tissue perfusion is challenging but necessary to diagnose peripheral vascular insufficiency. Because near infrared (NIR) radiation can penetrate relatively deep into tissue, significant attention has been given to intravital NIR fluorescence imaging.

**Methodology/Principal Findings:**

We developed a new optical imaging-based strategy for quantitative measurement of peripheral tissue perfusion by time-series analysis of local pharmacokinetics of the NIR fluorophore, indocyanine green (ICG). Time-series NIR fluorescence images were obtained after injecting ICG intravenously in a murine hindlimb ischemia model. Mathematical modeling and computational simulations were used for translating time-series ICG images into quantitative pixel perfusion rates and a perfusion map. We could successfully predict the prognosis of ischemic hindlimbs based on the perfusion profiles obtained immediately after surgery, which were dependent on the preexisting collaterals. This method also reflected increases in perfusion and improvements in prognosis of ischemic hindlimbs induced by treatment with vascular endothelial growth factor and COMP-angiopoietin-1.

**Conclusions/Significance:**

We propose that this novel NIR-imaging-based strategy is a powerful tool for biomedical studies related to the evaluation of therapeutic interventions directed at stimulating angiogenesis.

## Introduction

Peripheral tissues are vulnerable to necrosis under conditions that promote vascular insufficiency. Peripheral vascular insufficiencies are highly prevalent and usually result from diabetic complications or systemic atherosclerosis [1]–[3]. Perfusion imaging tools used to visualize the structure of the vasculature or functional perfusion levels are useful in preclinical drug discovery research and could have clinical applications [4], [5]. For these purposes, functional perfusion imaging is superior to structural vascular imaging because prognosis of vascular insufficiency is directly coupled to the functional perfusion level rather than to the vascular structure [6]. For example, highly varied outcomes of the ischemic hindlimbs might arise from differences in preexisting collaterals [7]. Therefore, measurements of the functional tissue perfusion through vessels including collaterals are required to evaluate vascular insufficiency and predict the prognosis. In addition, quantitative measurements of peripheral tissue perfusion are required to enable comparative studies, e.g., evaluating the effects of drugs *in vivo* or making clinical decisions based on patient information. Minimizing the invasiveness of perfusion imaging tools is a primary concern for clinical applications. Several methods have been used to quantify perfusion, including scintigraphic and positron emission tomography (PET) imaging and magnetic resonance imaging (MRI). However, these methods are too expensive for use in the diagnosis of tissue perfusion, especially for animal studies. The standard quantitative method for measuring perfusion in animals is microsphere perfusion; however, this method is suitable for *ex vivo*, rather than *in vivo*, measurements. Optical imaging, especially near infrared (NIR) fluorescence imaging, has proven effective for *in vivo* imaging of the vasculature and for estimation of functional perfusion [8].

For decades, indocyanine green (ICG) has been clinically used as a NIR fluorophore for intravital imaging, a marker for liver function [9], and a sensitizer for photodynamic therapy [10], [11]. For vascular imaging, ICG is FDA-approved, and NIR spectra enable deep tissue imaging [12], [13]. In addition, intravenously- injected ICG shows no extravasation except in abnormally permeable vasculature because ICG binds to the major serum protein, albumin [14]. Albumin-ICG complexes are segregated in the liver with first-order pharmacokinetics and excreted via the hepatobiliary pathway [15], [16]. Nevertheless, the rapid clearance of ICG has limited its use as an angiographic contrast medium. Recently, it was shown that the altered pharmacokinetics of ICG can be useful for estimating cerebral oxygenation and hemodynamics [17] and for measuring cerebral blood flow (CBF) [18]–[20]. Even though mathematical analysis was used to extract CBF from ICG dynamics in these studies, a lack of quantitative information limited the use of these techniques in the clinical setting [18]. The main purpose of our study was to develop a new method for functional and quantitative measurement of peripheral tissue perfusion and to demonstrate the feasibility of this method.

## Methods

### Murine hindlimb ischemia model

BalB/cAnNCriBgi-nu nude male mice were obtained from Charles River Japan Inc. (Yokohama, Japan). All mice were 7–8 weeks (15–20 g) of age at the time of study. Hindlimb ischemia was induced by ligation and excision of the right femoral artery and vein under ketamine–xylazine anesthesia. Animal care and experimental procedures were performed under the approval of the Animal Care Committees of KAIST.

For therapeutic angiogenesis studies, mice were divided into four groups after induction of ischemia for intramuscular injection with saline solution (20 µL with 0.9% NaCl and 0.1% bovine serum albumen (BSA)) alone as a control or saline solution containing COMP-angiopoietin-1 (cAng1) (20 µL of 200 ng/µL), vascular endothelial growth factor (VEGF) (20 µL of 130 ng/µL), or both cAng1 and VEGF (20 µL of 200 ng/µL cAng1 and 130 ng/µL VEGF). Serial ICG perfusion imaging was performed immediately after surgery and on postoperative day (POD) 3 and 7.

### NIR fluorescence imaging

We used two optical systems for NIR fluorescence imaging: Image station 4000 MM (Eastman Kodak Co., Rochester, NY, USA) and a customized system developed for this study. The customized imaging system employed a CCD digital camera (PIXIS 1024; Princeton instruments, Princeton, NJ, USA) with a custom-made 830-nm band-pass filter (Asahi Spectra USA, Torrance, CA, USA) and 760-nm light-emitting diode arrays (SMC760; Marubeni America, Sunnyvale, CA, USA).

For time-series ICG imaging, mice under ketamine–xylazine anesthesia were injected with an intravenous bolus injection of ICG (0.1 mL of 400 µmol/L; Sigma, St. Louis, MO, USA) into the tail vein. ICG fluorescence images were obtained for 12 min in 20-s intervals (Kodak imaging system) or in 1-s intervals (custom optical imaging system) immediately after injection. After the serial imaging, a silhouette image of the mouse was taken under white light to obtain the region of interest (ROI) mask of the ischemic and normal hindlimbs.

### *In silico* modeling

To identify the underlying relationships between the spatiotemporal ICG profiles and the perfusion rates in peripheral tissue, we performed an *in silico* study that included mathematical modeling and computational simulation analysis of ICG pharmacokinetics. To quantitatively represent tissue perfusion, perfusion rate (%/min) was defined as the fraction of blood exchanged per minute in the vascular volume of the ROI. We assumed that blood volume through the vasculature is time invariant during the imaging; therefore, volumetric inflow and outflow were thought to be the same (Figure S1A). To describe the regional ICG pharmacokinetics *in vivo*, the rear half of a mouse was divided into three compartments: the trunk, the normal hindlimb, and the ischemic hindlimb. Vascular input function (VIF) of ICG in normal tissues has been described as a uniexponential function of ICG excretion by the liver because liver dynamics dominate ICG excretion [16]. Therefore, ICG fluorescence intensity in normal tissues could be described by the following equations:
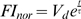
(1)and
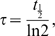
(2)where 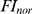 is the ICG fluorescence intensity in the VIF of normal tissues, *V_d_*, is the vascular density, t is time, 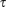 is the time constant for systemic dynamics, and t_1/2_ is the ICG half-life in the trunk.

The half-life of ICG decay varies, ranging from 3 to 5 min, according to idiosyncratic variations in hepatic function [9], anesthetizing conditions, and hemodynamic status. To normalize the variations in the excretion rate, we introduced ‘*τ*’ for all equations mentioned below.

The change in concentration of the ROI can be described using Fick's law:
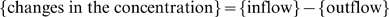
Therefore, the dynamics of ICG fluorescence intensity in the ischemic hindlimbs, *FI_isc_*, are a function of time and are represented by
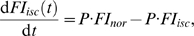
(3)where parameter ‘*P*’ represents the perfusion rate of the ischemic hindlimbs. The *FI_isc_* is defined as follows using Equations 1 and 3:
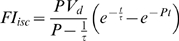
(4)Because the dynamics of ICG fluorescence intensity after tissue injection showed a bell-shaped curve, the differential value of *FI_isc_* is assigned as zero at the time-to-peak (*T_max_*). Therefore, the differential value of equation 4 is zero at the *T_max_*.
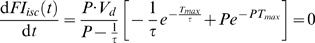
(5)

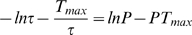
(6)


**Figure 1:**
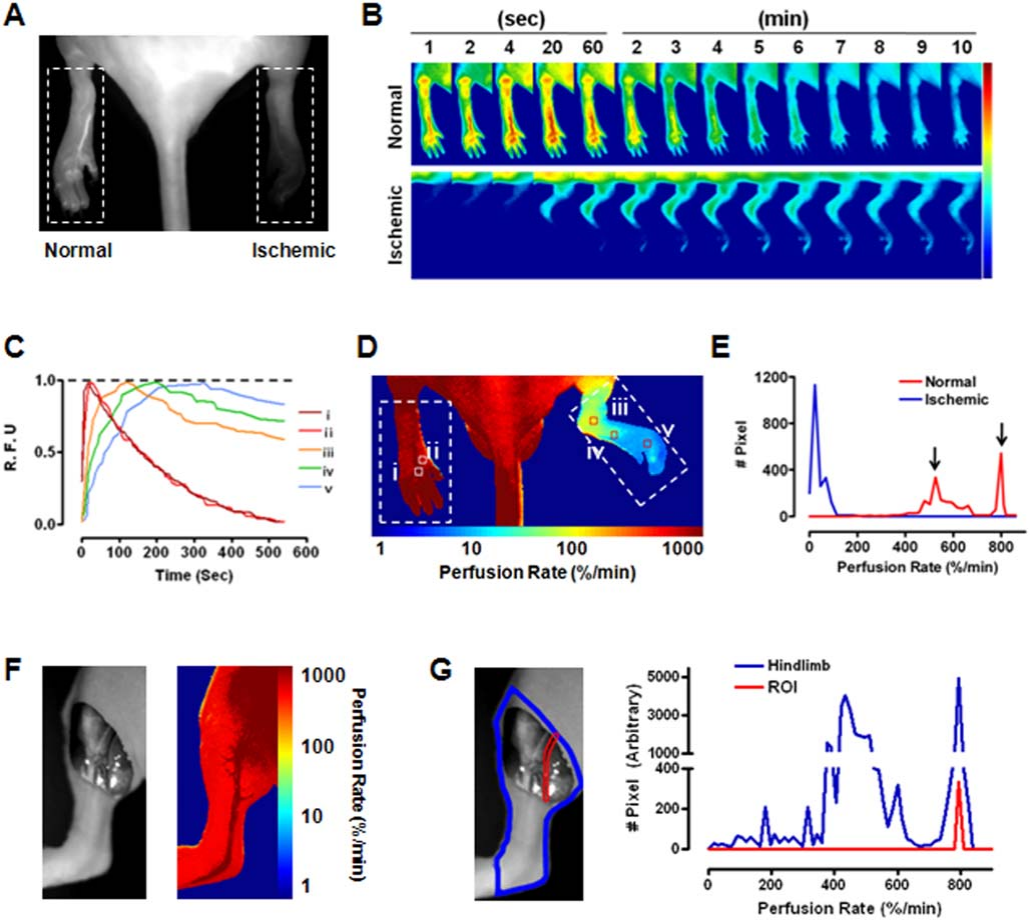
Quantitative measurement of perfusion rate based on spatiotemporal ICG dynamics. (A) Contrast NIR fluorescence angiography. (B) Temporal sequence of ICG fluorescence in the hindlimb ischemia model. The number indicates the time after ICG injection. (C) Temporal dynamics in the regions indicated with boxes in *D* are plotted. Relative fluorescence units (R.F.U.) were normalized to the maximal intensity. (D) Perfusion map reconstructed based on the time-series ICG images shown in *B*. (E) Histogram of perfusion rates in each limb for the regions indicated by the dotted box in *D*. The arrows indicate two perfusion rate peaks in the normal limb. (F) Photographic image of a normal hindlimb after skin excision to expose femoral vessels (right). Perfusion map of the limb (left). (H) Histogram of perfusion rates of the total hindlimb is indicated by the blue line, and the red line depicts the perfusion rates of the ROIs marked by the red line on femoral vessels.

From equation 5, we could derive equation 6 and calculate the perfusion rate (*P*) of each pixel in the ROI using two parameters, the *T_max_* of each pixel and ICG half-life in the trunk. The simulation for the temporal ICG dynamics was run with different perfusion rate values (Figure S1B), which was consistent with actual ICG dynamics (see Figure 1C). The relationships among the ICG half-life, the time-to-peak, and the perfusion rate are shown in Figure S1C and D.

### Image analysis

ROI masks of ischemic and normal limbs were drawn on the silhouette image to extract regions corresponding to limbs in the acquired time-series NIR fluorescence images. Likewise, the square mask was also drawn on the trunk region to obtain parameter 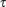 for calculating the systemic half-life of ICG. Image processing and data analysis were performed using Visual C^++^ (version 6.0; Microsoft, Redmond, WA, USA) and Matlab (MathWorks, Natick, MA, USA).

The half–life of ICG was measured in the square mask region on the trunk and the time-to-peak of each pixel was determined to obtain the perfusion rate of a pixel in the ROI. The pixel number of a ROI in a limb was typically around 1200–1500. The perfusion rate in each pixel was then calculated using the equation derived by modeling. The result was visualized as a pseudocolor-coded perfusion map or a histogram.

To determine the probability of necrosis, necrotic regions in the ischemic limbs were assessed on POD 7. Perfusion rates that were measured 4 h post-op were analyzed in both the necrotic and non-necrotic regions. The correlation between the regional perfusion rate (average perfusion rate of 2×2 pixels) and the necrosis of this region was calculated. The relationship between necrosis determined at POD 7 and the regional perfusion rate measured immediately after surgery was obtained based on data from 20 mice. A necrosis map was generated by mapping the predictive necrosis of individual regions as a color-coded picture.

### Histological analysis

Vessel density within the calf muscles of the ischemic limbs was quantified by histological analysis. The ischemic muscles were perfused with 4% (w/v) paraformaldehyde (Sigma) and embedded in paraffin. Calf muscle sections (10 µm thickness) were stained with hematoxylin–eosin (H&E) and also double-stained with anti-CD31 antibody (Chemicon, Temecula, CA, USA) and a monoclonal anti-α-smooth muscle-actin (α-SMA) antibody (Sigma) conjugated to fluorescein isothiocyanate (FITC). Proteins immunoreactive with the anti-CD31 antibody were stained with a hamster anti-mouse IgG antibody conjugated with rhodamine (Jackson Laboratories, West Grove, PA, USA). The stained sections were visualized by confocal microscopy (Axiovert LSM 510 META; Zeiss, Oberkochen, Germany). Vessel density was expressed as the number of α-SMA and CD31 double-positive micro- (16–63 µm in diameter) and macrovessels (>63 µm in diameter) per high-power field (magnification×400) [21].

### Laser Doppler imaging (LDI)

Mice were scanned using LDI (MLDI5063; Moor Instruments Ltd., Devon, UK) after ICG perfusion imaging at POD 0. Three consecutive scans were performed until blood flow measurements were stable. The images were subjected to computer-assisted quantification of blood flow.

### Micro-CT angiography

Mice were placed in an induction chamber with 4% isofluorane in oxygen to induce anesthesia. After anesthetization, mice were injected with PEG-conjugated gold nanoparticles (400 µL of 0.2 mol/L) intravenously and placed on a volumetric CT scanner (NFR-MXSCAN-G90; NanoFocusRay, Iksan, Korea) [22]. While scanning, the mice remained anesthetized under 1.5% isofluorane in oxygen. In total, 600 images were acquired at 65 kVp, 55 µA, and 800 ms per frame. Images were reconstructed using the Feldkamp cone-beam reconstruction algorithm. The reconstruction image size was 1024×1024 pixels, and 512 slices were acquired. The final reconstructed data were converted to the Digital Imaging and Communications in Medicine (DICOM) format to make three-dimensional (3-D)-rendered images using 3-D-rendering software (Lucion; MeviSYS, Seoul, Korea).

### Statistical analysis

Data are expressed as means±SEM. Statistical significance was assessed by Student's two-tailed t-test for two groups or by one-way ANOVA and Bonferroni *post hoc* test for three or more groups.

## Results

### Estimation of tissue perfusion based on time-series analysis of ICG pharmacokinetics

The feasibility of NIR fluorescence imaging [8] was examined to monitor peripheral tissue perfusion using albumin microspheres conjugated with the NIR fluorophore ICG (Method S1) [23]. Contrast NIR fluorescence angiography showed obvious differences in maximal fluorescence intensity between normal and ischemic hindlimbs (Figure 1A). Even though contrast optical imaging distinguished well-perfused from hypoperfused tissues, it only provided qualitative information. Because the local concentration of ICG is dependent on regional tissue perfusion, we measured the spatiotemporal dynamics of ICG pharmacokinetics by obtaining time-series images of free ICG fluorescence [17]. As expected, ICG dynamics in normal hindlimbs showed a rapid time-to-peak of around 20 s after ICG injection and an exponential decay of ICG fluorescence (Figure 1B, upper panel). In contrast, ischemic limbs showed markedly altered dynamics of ICG fluorescence compared to controls and a remarkably delayed time-to-peak and clearance (Figure 1B, lower panel). We also observed considerable differences in the ICG dynamics between different regions in the same ischemic hindlimb (Figure 1C), suggesting spatial heterogeneity of perfusion in the peripheral tissue. Based on the equation obtained *in silico*, we translated the temporal ICG dynamics of each pixel into the perfusion rate and constructed a perfusion map (Figure 1D). This perfusion map showed the spatial distribution of perfusion rates with high spatial resolution. Histogram analysis showed a bimodal distribution of perfusion rates in the normal limb, while only one peak with a lower perfusion rate was observed in the ischemic hindlimb (Figure 1E). The two peaks of perfusion in the normal hindlimb appeared to correspond to macrovasculature regions with high perfusion rates and microvascular compartments with lower perfusion rates. To confirm whether the right peak of the bimodal distribution corresponded to macrovascular regions, we removed the skin of the calf to reveal the femoral artery and vein and performed ICG perfusion imaging (Figure 1F). The exposed femoral artery and vein were distinguishable with higher perfusion rates compared to the surrounding area in the perfusion map (Figure 1F, right panel). Moreover, the perfusion rates of the ROI corresponding to the exposed macrovascular regions matched the right peak of the bimodal distribution in the histogram of the normal limb (Figure 1G). Therefore, the histogram analysis of perfusion clearly indicated that the induction of ischemia abolished macrovasculature perfusion and significantly reduced microvascular perfusion.

### Functional levels of perfusion by preexisting collaterals determine the prognosis of hindlimbs after induction of ischemia

**Figure 2:**
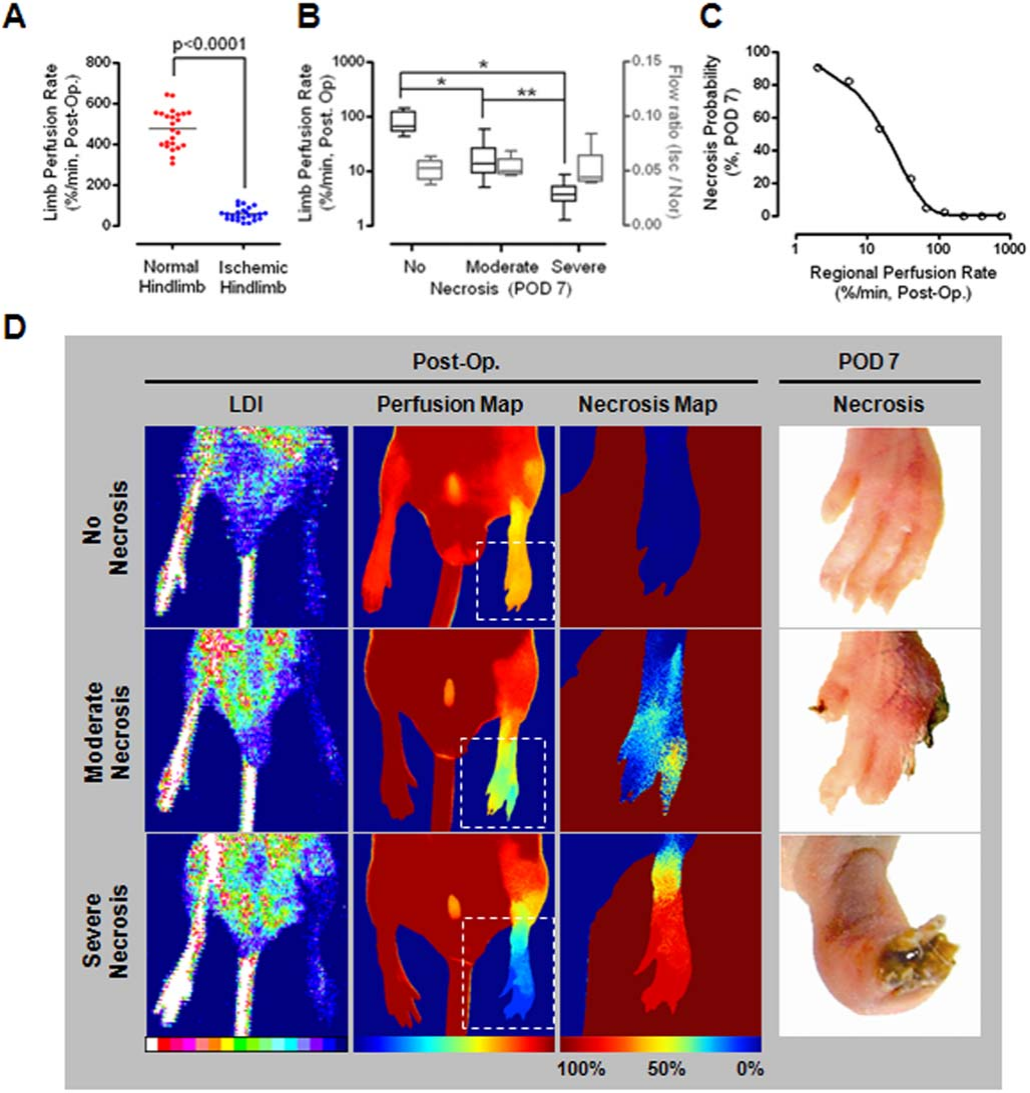
Necrosis predictability of ICG dynamic perfusion imaging. (A) Average limb perfusion rates of the normal and ischemic hindlimbs at POD 0 (postoperative day 0; Post-Op.). The gray lines indicate the mean of each group. The *P* value is shown (Student's t-test, n = 24). (B) Correlation between average limb perfusion rates in the ischemic limbs at POD 0 (black boxes and whiskers) and limb necrosis levels at POD 7. Bonferroni *post hoc* test applied to the significant effect of groups (ANOVA *F*_2,44_ = 58.358, *P*<0.001). *, *P*<0.001. **, *P* = 0.054. The *y*-axis is in log scale. The gray boxes and whiskers show a relationship between the ischemic/normal laser Doppler blood flow ratio at POD 0 and limb necrosis levels at POD 7. (ANOVA *F*_2,16_ = 0.465, *P* = 0.636). (C) Sigmoidal relationship between regional perfusion rates of the ischemic hindlimbs at POD 0 and necrosis probability of the corresponding region at POD 7. Graphs were drawn from data of 70,714 regions from 20 mice (Boltzmann sigmoidal fit, R^2^ = 0.998, *P*<0.001). The *x*-axis is in log scale. (D) Comparison of diagnostic predictability between LDI and ICG perfusion imaging. For three representative mice with different prognoses, LDI images, perfusion maps, and necrosis maps at POD 0 are shown along with pictures of the ischemic limbs at POD 7. The region for the necrosis map from the corresponding area in the perfusion map is indicated by the white dotted boxes.

To validate ICG dynamic perfusion imaging analysis, we estimated the tissue perfusion immediately after induction of ischemia (Figure 2A). Normal hindlimbs demonstrated a perfusion rate of 475±19%/min, while the perfusion rate of ischemic hindlimbs was 56±6%/min (12.2% of the normal hindlimbs). Considerable variability was observed in the outcome of the ischemic hindlimbs, which might have arisen from differences in preexisting collaterals [7]. To validate our method, we investigated the correlation between the initial perfusion rates of the ischemic tissues and the prognosis, both of which are dependent on preexisting collaterals. The animals were classified into three groups according to the severity score of limb necrosis (score 0 = no necrosis; score 1 = toe necrosis; score 2 = foot necrosis; score 3 = ankle necrosis; score 4 = autoamputation of the entire leg. Moderate necrosis includes scores 1 and 2 and severe necrosis includes scores 4 and 5.), which was determined by visual inspection on POD 7. These groups included no necrosis (16%), moderate necrosis (40%), and severe necrosis (46%; Figure S2). We then compared the perfusion rates of these three groups from the ischemic limbs immediately after surgery (Figure 2B). In 16% of the animals with the best prognosis, the perfusion rate of the ischemic hindlimbs was significantly higher compared to other groups; the average perfusion rates of the groups with moderate and severe necrosis were 19.4 and 4.11%/min, respectively (Figure 2B). However, the representative data of the ischemic/normal blood flow ratio using LDI did not provide enough sensitivity to show correlations between the initial blood flow and the necrosis level in the ischemic limbs. Since the preexisting vasculature appeared to contribute significantly to the prognosis, we questioned whether the perfusion map obtained immediately after surgery could precisely predict future necrosis. To address this, we determined the relationship between the probability of regional tissue necrosis on POD 7 and the given perfusion rate of the corresponding region after operation (Figure 2C). This relationship was expressed in an inverse sigmoidal function. By mapping perfusion rates with necrosis probability, we generated a color-coded necrosis map to display the spatial distribution of necrosis probability on POD 7. Comparisons between the predicted levels of necrosis to the actual necrosis levels in ischemic limbs verified the feasibility and accuracy of the necrosis map. The perfusion maps of representative mice showed considerable contrasts in ischemic limb perfusion rates, and the necrosis maps showed a remarkably high correlation with the actual necrotic regions that were determined on POD 7 (Figure 2D). To further validate the sensitivity of our newly developed tool using ICG dynamic imaging, we compared the perfusion map to conventional LDI, the current “gold standard” for *in vivo* blood flow measurements (Figure 2D, left panel) [24]. The LDI images of the three representative mice could only demonstrate overall qualitative decreases in the levels of perfusion in the ischemic limbs and could not, on a more quantitative level, differentiate between levels of perfusion (Figure S3). These results collectively indicate the superior sensitivity of ICG dynamic optical imaging, which is sensitive enough to predict future necrosis of ischemic tissues.

### Vascular endothelial growth factor and COMP-ang1 synergistically improve perfusion and subsequent prognosis of ischemic hindlimbs

**Figure 3:**
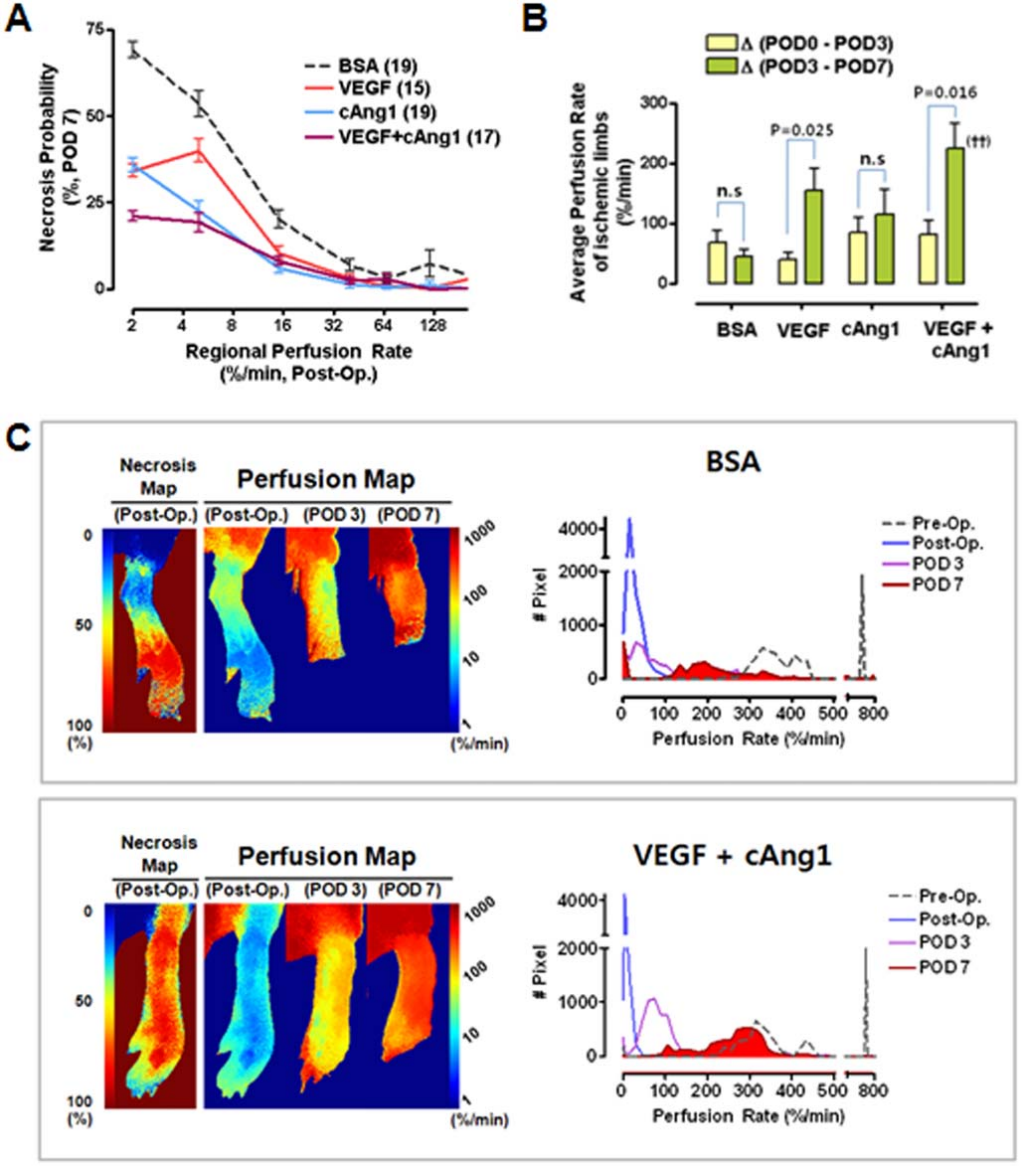
Synergistic proangiogenic effects of VEGF and cAng1. (A) Correlations between necrosis probability and regional perfusion rates were determined. (B) Differences in perfusion rates according to the time period are indicated for each group. ANOVA and Bonferroni *post hoc* test applied to the significant effect of groups on Δ (POD 3 - POD 7), (ANOVA *F*_3,33_ = 4.890, *P* = 0.006). ^††^, *P* = 0.004 vs. BSA control. Paired t-tests were performed for each intragroup comparison. (C) Representative examples of BSA control and combined treatment groups.

For therapeutic angiogenesis studies, 70 mice were divided into four groups. The initial perfusion rates of the ischemic limbs were not significantly different among groups (ANOVA, F_3,33_ = 0.667, p = 0.578, Figure S4). After intramuscular injection of angiogenic factors in the ischemic limbs, follow-up ICG perfusion imaging was serially performed on POD 3 and 7. As expected, the probability of necrosis in regions with low perfusion rates (2–60%/min) was significantly improved by treatment with VEGF, cAng1, or both (Figure 3A). Intergroup comparisons of time-dependent increments in perfusion rates clearly demonstrated synergistic therapeutic effects of VEGF and cAng1, especially between POD 3 and 7 (Figure 3B and Figure S5); this was consistent with results from our previous study [25]. The representative comparison shows that combined treatment with VEGF and cAng1 improved perfusion and the subsequent prognosis of the ischemic hindlimbs (Figure 3C). In the control animals, the amount of necrotic tissue accurately matched the necrosis predicted by postoperative (post-op) perfusion measurements. Slightly diminished perfusion was initially observed in the combined treatment animal, which was predicted to have a poorer prognosis compared to the control; however, the final outcome was much better than that of the control, indicating a protective effect of combined treatment. Histogram analysis showed that the unimodal peak shifted to the right after surgery and was almost completely restored to the level of the normal hindlimb, suggesting a dominant effect of proangiogenic factors on the microvasculature.

### Arteriogenesis partly mediates improvement of tissue perfusion

**Figure 4:**
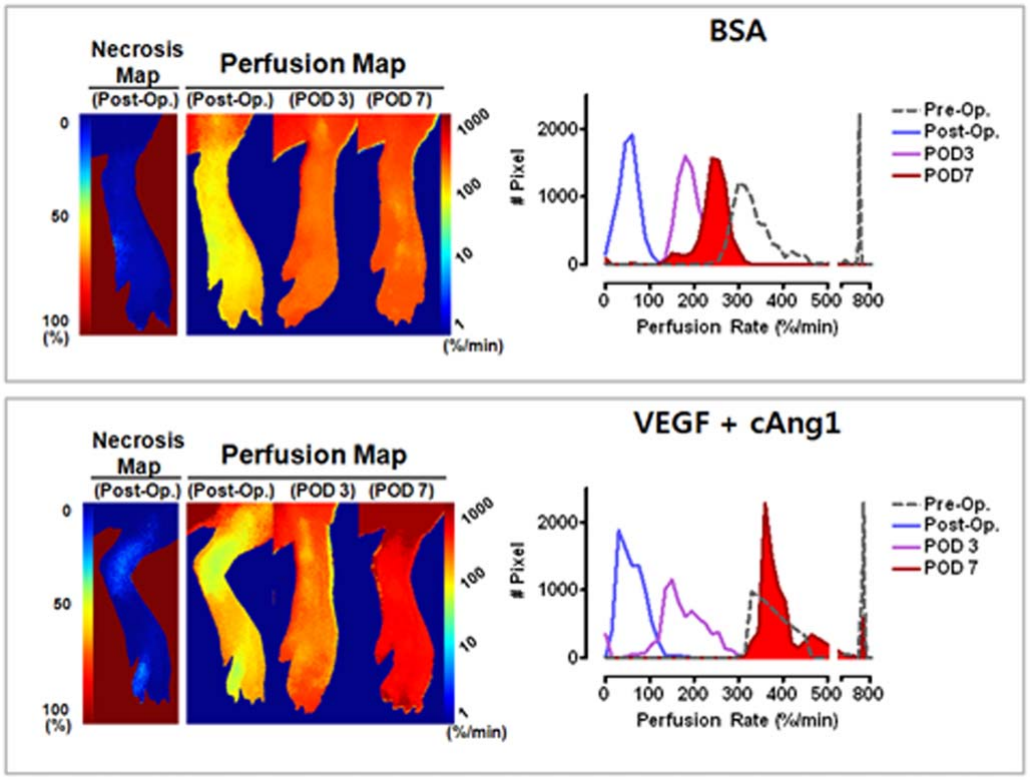
Therapeutic effects of VEGF and cAng1 on tissue perfusion. Representative cases of BSA control and combined treatment groups.

**Figure 5:**
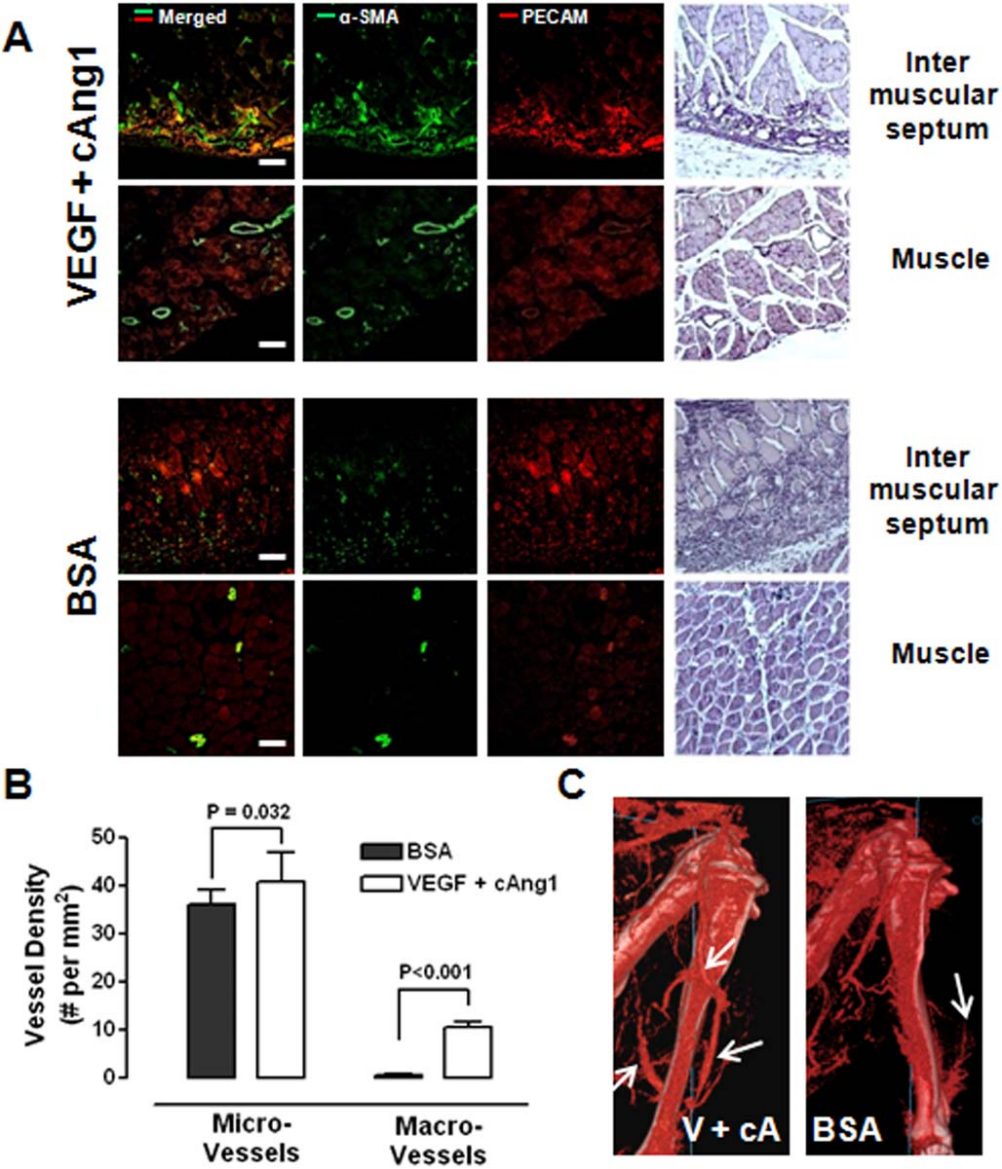
Arteriogenic effects of VEGF and cAng1. (A) Confocal micrographs of calf muscle sections from ischemic limbs treated with VEGF/cAng1 and BSA. Magnification, 200×. Bar = 100 µm. (B) Macro- and microvessel densities were evaluated in the calf muscles. The number of vessels was expressed as vessels/mm^2^. Data from six fields of the four samples were averaged. Student's t-test was applied. (C) Micro-CT angiography. The arrows indicate macrovessels in the calf.

As previously indicated, some animals never developed tissue necrosis even though identical surgical protocols to induce ischemia were used. Therefore, uneven distribution of such nonresponsive animals into the study groups can severely distort the results [26]. Since our method can quantitatively determine the efficacy of the treatment on the perfusion rate rather than grading visible necrosis, we tested whether our method was also useful for evaluating the efficacy of the proangiogenic factors in this nonresponsive group (Figure 4). The perfusion rate of a representative case in the control group at post-op was measured as 56%/min; hence, the ischemic limb was predicted to be salvaged. The limb perfusion rate increased to 183%/min on POD 3 and 227%/min on POD 7 (Figure 4, upper left panel). The histogram analysis of the ischemic limb also showed a gradual time-dependent shift to the right; however, the histogram of perfusion rates on POD 7 showed incomplete recovery when compared to the pre-op level (Figure 4, upper right panel). In a representative case in the combination treatment group, the post-op limb perfusion rate was estimated to be 68%/min, and no necrosis was predicted. The limb perfusion rate increased to 161%/min by POD 3 and increased to 364%/min by POD 7 (Figure 4, lower left panel). The histogram showed almost complete recovery of perfusion by POD 7 when compared to the initial level of perfusion before surgery; notably, there was a recovery of macrovascular conductance on POD 7 even though the level was lower than that of the basal level, suggesting regeneration of the macrovasculature by combined treatment of VEGF and cAng1 (Figure 4, lower right panel). Likewise, the postmortem histological study demonstrated that PECAM and α-SMA double-positive vessels increased significantly in number and size in the intermuscular septa and muscle fibers in the combined treatment group compared to the control group (Figure 5A). Quantitative measurements showed a 19.7-fold increase in macrovascular density, while the combined treatment increased the density of both the macro- and microvessels (Figure 5B). Moreover, micro-CT scans depicted more than three macrovessels (>150 µm in diameter) in the calf of the treated animals on POD 7 (Figure 5C, left panel); there were only thin macrovessels (smaller than 100 µm) in the calf of the control animals (Figure 5C, right panel). Collectively, these results suggest that combined treatment with VEGF and cAng1 increases perfusion and improves the outcome of the ischemic hindlimbs partly via induction of arteriogenesis and microvascular angiogenesis.

## Discussion

In this paper, we propose a novel NIR imaging method for the quantitative measurement of perfusion in peripheral tissues using planar reflectance imaging, which is simpler to implement compared to the tomographic method, by introducing mathematical modeling and a computational simulation. The validity of this novel method was evaluated using a murine model of hindlimb ischemia and therapeutic interventions directed at stimulation of angiogenesis. We showed that time-series imaging of intravenously injected ICG can provide quantitative information regarding peripheral tissue perfusion precisely enough to predict the prognosis of the ischemic tissues and to verify the therapeutic effects of proangiogenic factors such as VEGF and cAng1. For analysis of ICG dynamics, we designed a biologically meaningful mathematical model.

Previous studies that used ICG dynamics to estimate blood perfusion failed to provide quantitative information that was required for interindividual comparison. In those studies, blood perfusion was estimated based on the maximal fluorescence intensities of injected ICG, which depend heavily on the heterogenous optical properties of regional tissues [17], [18]. Moreover, static reflectance images from different animals or from the same animal at different time points are generally insufficient for yielding quantitative insights, because the fluorescence intensity depends on the depth of the signal and the volume and optical properties of the tissue. Thus, we performed time-series NIR fluorescence imaging to obtain the spatiotemporal dynamics of ICG and translated the time-series images into quantitative perfusion maps. Furthermore, we compensated for idiosyncratic variations in ICG pharmacokinetics to obtain quantitative information on perfusion rates, because the idiosyncratic variation of ICG dynamics depends on the level of anesthesia, systemic hemodynamic properties, and variations in hepatic clearance that can affect the evaluation of perfusion rate. This quantitative information enabled us to predict the necrotic profile of ischemic tissues.

The necrosis profiles of the ischemic limbs were variable among subjects even when the same surgery protocol and the same strain of animals were used [26]. Necrosis profiles of the ischemic hindlimbs are considered to be determined by two main factors: the preexisting collateral circulation [7] and vascular genesis [27], [28]. Variability of necrosis profiles between subjects could be caused primarily by differences in the preexisting collateral circulation, because the substrates of vascular genesis are preexisting collateral arteries [29]. Therefore, we made two predictions. First, if the perfusion rate measured at post-op represents the preexisting collateral circulation, we predicted that this would show a significant relationship with future necrosis of the ischemic limb. As expected, the regional perfusion rate showed a significant inverse (sigmoidal shape) relationship with the probability of necrosis of the region. Second, we predicted that aiding angiogenesis by treatment with proangiogenic factors would alter the necrosis profile based on the preexisting collateral circulation. Significantly, treatment with the proangiogenic factors VEGF and cAng1 improved the necrosis profile.

Our method has several advantages for analyzing the perfusion and prediction of necrosis compared to conventional methods. First, quantitative measurement of tissue perfusion can allow interindividual comparative analysis of tissue perfusion. Second, this highly sensitive and quantitative measurement of perfusion can be used to equally distribute ischemic hindlimb model mice in control and experimental groups, which would lessen data distortion and decrease the variability of the experimental results. We have observed that the prognosis of ischemic tissues from animals subjected to identical surgical protocols varies considerably. This results in intrinsic data heterogeneity, which could severely misrepresent the outcome of drug interventions. Third, changes in perfusion due to proangiogenic factors in ischemic tissues can be evaluated and arteriogenic processes can be differentiated from angiogenesis because our method can classify conductance through the macrovasculature. We observed that combined treatment with VEGF and cAng1 restored the bimodal histogram perfusion rate pattern in the ischemic limbs, reflecting the emergence of macrovascular components. Histological and CT-angiogram analysis also showed that combined treatment with VEGF and cAng1 induced arteriogenesis along with angiogenesis. These results suggest that ICG dynamic perfusion imaging might be applied to differentiate macroangiopathy from microangiopathy.

Our method has several potential limitations as well. First, induction of ischemia transiently changes vascular permeability and dilatation, which play roles in functional recovery from acute hindlimb ischemia [30]. Our method might underestimate perfusion rates in cases of increased vascular permeability because delayed washout kinetics of ICG as a result of leakage are typically interpreted as decreased perfusion. Moreover, our method cannot distinguish between perfusion changes due to blood flow versus microvessel density. Therefore, it is important to control the physiologic factors that can affect blood flow such as blood pressure, pulse rate, and body temperature for consistent measurement of tissue perfusion. Second, even though NIR can penetrate biological tissues deeply, the depth of NIR penetration nonetheless restricts measurement of perfusion to peripheral tissues. For analysis of deep-seated organs, endoscope-coupled technology might help to overcome this limitation. Finally, it is technically difficult to estimate accurate perfusion rates for normal tissues, especially in the case of normal tissues that have very fast ICG kinetics. For accurate measurements of such fast kinetics, the images with maximal intensity should be captured, which correspond to images of the time-to-peak. Since the 20 s time-series imaging of the Kodak imaging system is too long an interval to capture the time-to-peak of normal limbs, we developed a customized NIR fluorescence imaging system that can shorten the interval to as little as 1 s. In this way, we were able to capture the peak intensity successfully even in normal limbs. In addition we confirmed that these two imaging methods provided basically the same results when estimating perfusion rates in ischemic limbs, which showed slower ICG kinetics.

In summary, our results indicate that ICG dynamic perfusion imaging can quantitatively evaluate perfusion in peripheral tissues. Moreover, this imaging technology could be used to predict future necrosis profiles and evaluate angiogenesis. ICG perfusion imaging can be a cost- and time-efficient tool for diagnosis of patients with peripheral vascular insufficiencies. Although further studies evaluating ICG imaging for clinical applications are needed, we believe that this revolutionary imaging method may provide new impulse to the multidisciplinary analysis of biomedical imaging and may contribute to the development of clinical diagnostic techniques for peripheral vascular disease.

## Supporting Information

Method S1 Contrast NIR fluorescence angiography
(0.02 MB DOC)

Figure S1 (A) Schematic model diagram for in silico analysis: simplified model for local tissue perfusion including volumetric inflow (*v_i_*), volumetric outflow (*v_o_*), and blood volume of the vasculature (*V_v_*). (B) Simulated results for temporal ICG dynamics with different perfusion rates. Maximal intensity has been normalized. R.F.U., relative fluorescence units. (C) Simulated ICG dynamics with different perfusion rate values and half-lives of ICG. (D) Two parameters that determine the decay phase in ICG dynamics. The graph shows that both the half-life of ICG and the perfusion rate contribute to the time-to-peak of ICG dynamics.
(1.37 MB TIF)

Figure S2 Natural course of ischemic limbs. Severity score of ischemic hindlimb necrosis was assessed using the following scale: score 0 = no necrosis; score 1 = toe necrosis; score 2 = foot necrosis; score 3 = ankle necrosis; score 4 = autoamputation of the entire leg. Moderate necrosis includes scores 1 and 2 and severe necrosis includes scores 4 and 5. The prognosis of the ischemic hindlimbs of each group was plotted with the severity score over time. Note that tissue necrosis occurred mainly within 7 days after surgery.
(0.36 MB TIF)

Figure S3 Comparison of the tissue perfusion from a collection of ROIs between the ICG perfusion imaging and LDI. Note the LDI cannot differentiate between the levels of perfusion especially in the low perfusion section, from 1% to 100%/min. In this section, the broad range of the necrosis probability was distributed from 0% to 90%.
(0.16 MB TIF)

Figure S4 Post-operative perfusion rates in the ischemic limbs of four groups of mice that were distributed evenly for therapeutic angiogenesis study. Note the non-significant difference in perfusion rates at post-op among the groups [ANOVA, F_(3,33)_ = 0.667, p = 0.578, Bonferroni Post hoc: p = 1 among all groups].
(0.16 MB TIF)

Figure S5 Each line indicates the time-dependent change of the average perfusion rate of ischemic hindlimbs from each mouse.
(0.33 MB TIF)
