# Supplementary material for: Quantitative Analysis of Peripheral Tissue Perfusion Using Spatiotemporal Molecular Dynamics
Source: PLoS One. 2009 Jan 26;4(1):e4275. doi: 10.1371/journal.pone.0004275 (PMC2626246; doi:10.1371/journal.pone.0004275)
Supplement: Method S1 — Contrast NIR fluorescence angiography (0.02 MB DOC) [file pone.0004275.s001.doc]

**Supporting Methods**

**Method S1. Contrast NIR fluorescence angiography**

As illustrated in Figure 1A, for contrast NIR fluorescence angiography we used albumin microsphere-conjugated ICG, since tissue perfusion can be measured using either magnetic resonance imaging (MRI) or ultrasound-based imaging using albumin microspheres.[1] In addition, the myocardial blood flow (MBF) estimated *in vivo* using MRI and albumin microspheres agreed with the MBF measured *in vitro* using radioisotope-labeled microspheres within the 95% confidence intervals.[2] Therefore, albumin microsphere-ICG complexes were used as the contrast medium for *in vivo* contrast NIR fluorescence angiography to estimate tissue perfusion, because ICG is an NIR fluorophore. The albumin microspheres were prepared as described previously.[3] Briefly, a 1:1:1 mixture of 5% dextrose, 5% albumin, and perfluorocarbon gas was sonicated for 60 s using an electromechanical sonicator (VCX750, Sonics & Materials, CT, USA). Then, 1 mL of perfluorocarbon-exposed sonicated dextrose albumin (PESDA) was mixed with 0.1 mL of 8 mmol/L ICG to make the albumin microsphere-conjugated ICG. This mixture was then filtered through a 0.2-µm pore filter to exclude any free ICG. Then, 0.1 mL of the albumin microsphere-conjugated ICG was injected through the tail vein as contrast medium. Immediately after the injection, contrast-enhanced NIR fluorescence angiography was performed with 40-s exposures using a Kodak imaging system. Free ICG was used for time-series NIR fluorescence imaging and analysis of ICG dynamics.

**References**

1. Vogel R, Indermuhle A, Reinhardt J, Meier P, Siegrist PT, et al. (2005) The quantification of absolute myocardial perfusion in humans by contrast echocardiography: algorithm and validation. J Am Coll Cardiol 45: 754-762.

2. Jerosch-Herold M, Hu X, Murthy NS, Rickers C, Stillman AE (2003) Magnetic resonance imaging of myocardial contrast enhancement with MS-325 and its relation to myocardial blood flow and the perfusion reserve. J Magn Reson Imaging 18: 544-554.

3. Porter TR, Xie F (1995) Visually discernible myocardial echocardiographic contrast after intravenous injection of sonicated dextrose albumin microbubbles containing high molecular weight, less soluble gases. J Am Coll Cardiol 25: 509-515.
