# Supplementary figures and images for: Quantitative Analysis of Peripheral Tissue Perfusion Using Spatiotemporal Molecular Dynamics
Source: PLoS One. 2009 Jan 26;4(1):e4275. doi: 10.1371/journal.pone.0004275 (PMC2626246; doi:10.1371/journal.pone.0004275)

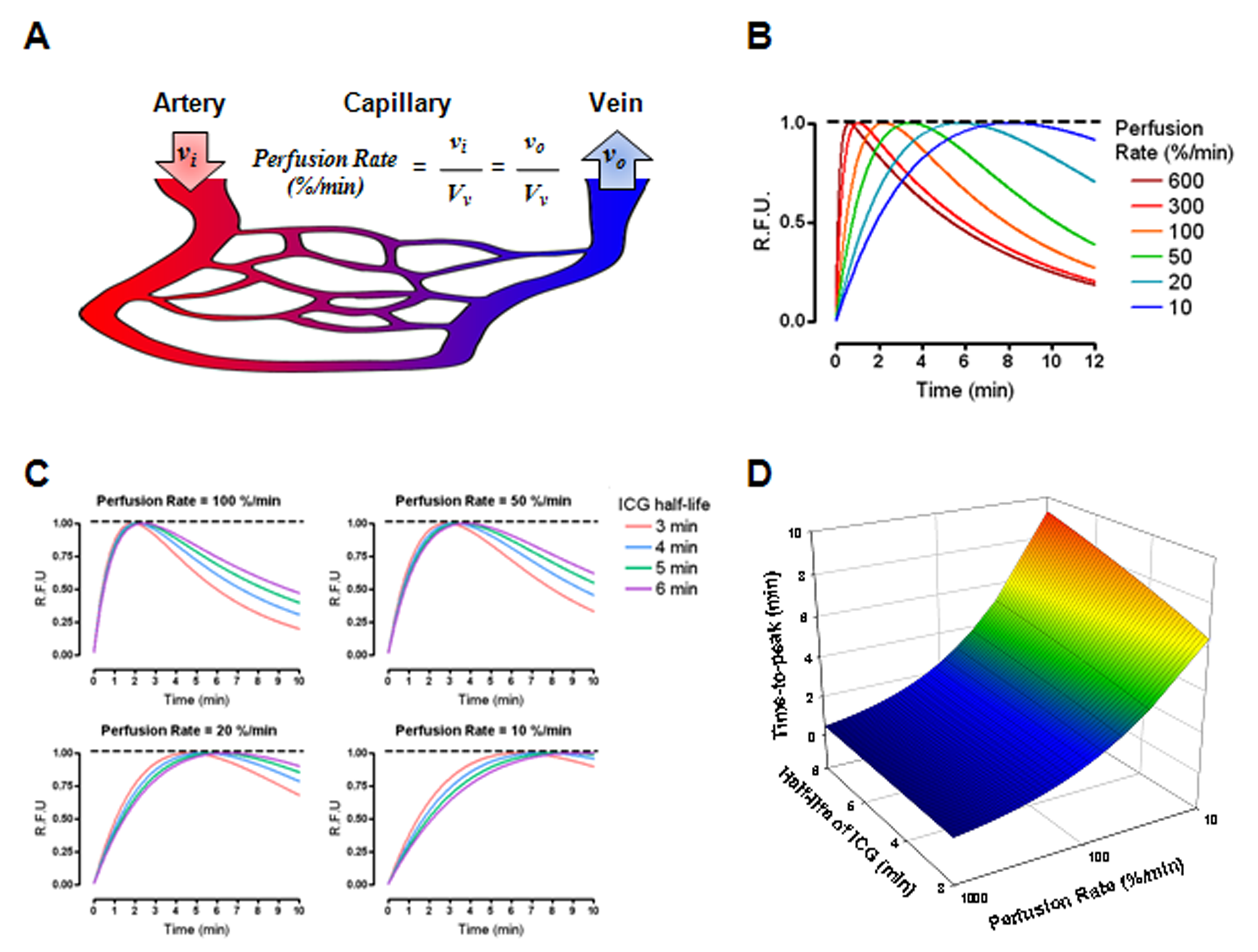

Supplement: Figure S1 — (A) Schematic model diagram for in silico analysis: simplified model for local tissue perfusion including volumetric inflow (vi), volumetric outflow (vo), and blood volume of the vasculature (Vv). (B) Simulated results for temporal ICG dynamics with different perfusion rates. Maximal intensity has been normalized. R.F.U., relative fluorescence units. (C) Simulated ICG dynamics with different perfusion rate values and half-lives of ICG. (D) Two parameters that determine the decay phase in ICG dynamics. The graph shows that both the half-life of ICG and the perfusion rate contribute to the time-to-peak of ICG dynamics. (1.37 MB TIF) [file pone.0004275.s002.tif]

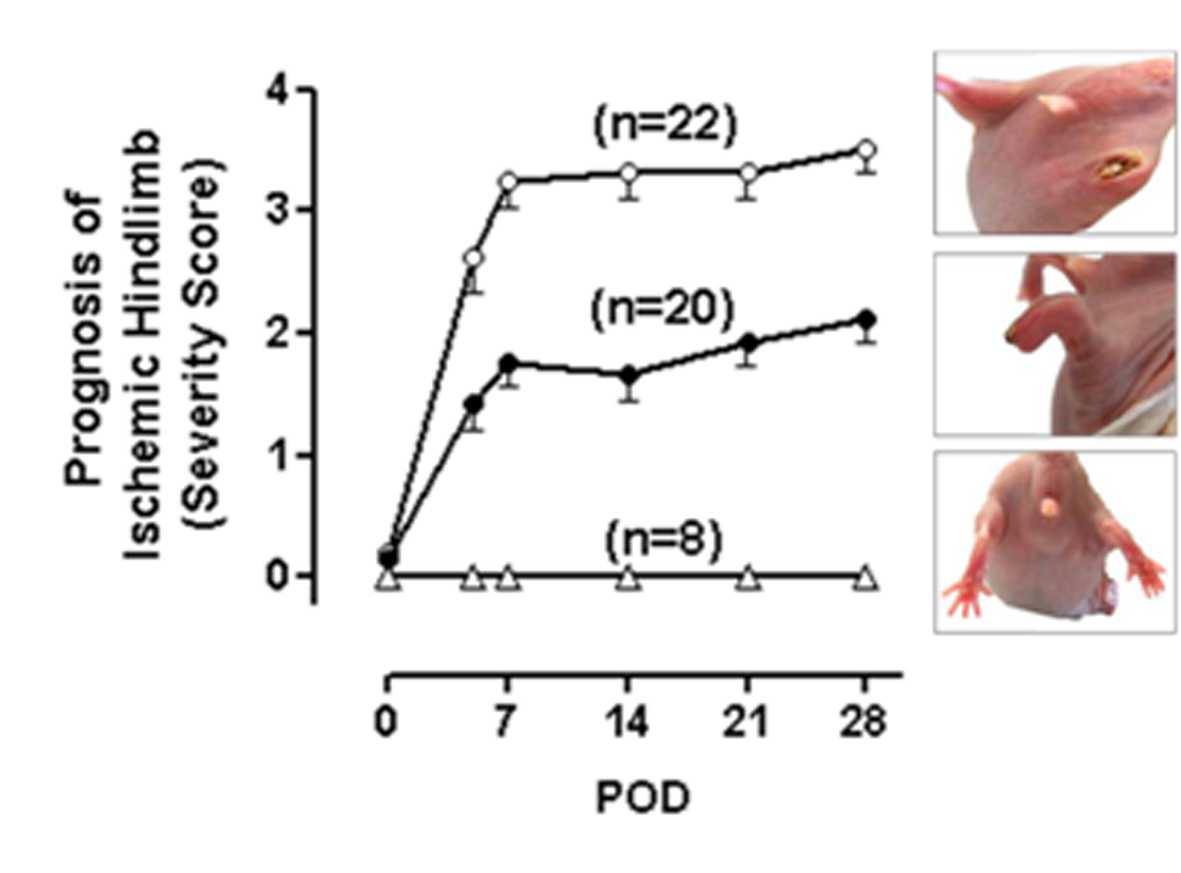

Supplement: Figure S2 — Natural course of ischemic limbs. Severity score of ischemic hindlimb necrosis was assessed using the following scale: score 0 = no necrosis; score 1 = toe necrosis; score 2 = foot necrosis; score 3 = ankle necrosis; score 4 = autoamputation of the entire leg. Moderate necrosis includes scores 1 and 2 and severe necrosis includes scores 4 and 5. The prognosis of the ischemic hindlimbs of each group was plotted with the severity score over time. Note that tissue necrosis occurred mainly within 7 days after surgery. (0.36 MB TIF) [file pone.0004275.s003.tif]

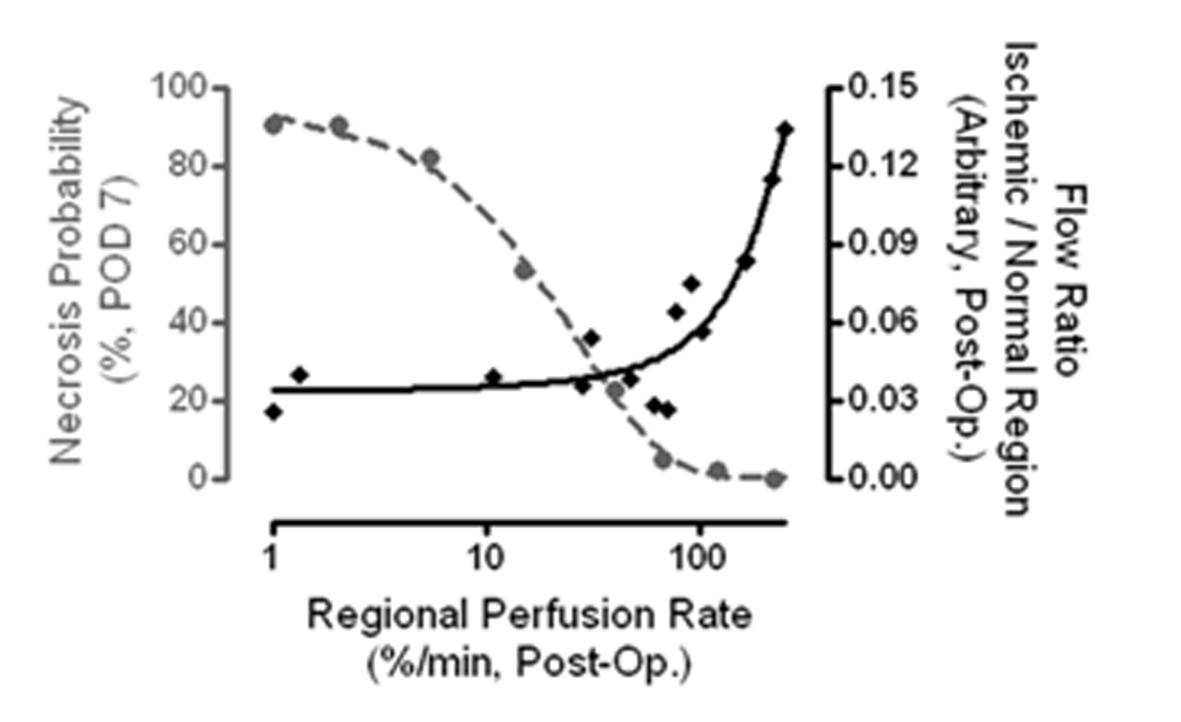

Supplement: Figure S3 — Comparison of the tissue perfusion from a collection of ROIs between the ICG perfusion imaging and LDI. Note the LDI cannot differentiate between the levels of perfusion especially in the low perfusion section, from 1% to 100%/min. In this section, the broad range of the necrosis probability was distributed from 0% to 90%. (0.16 MB TIF) [file pone.0004275.s004.tif]

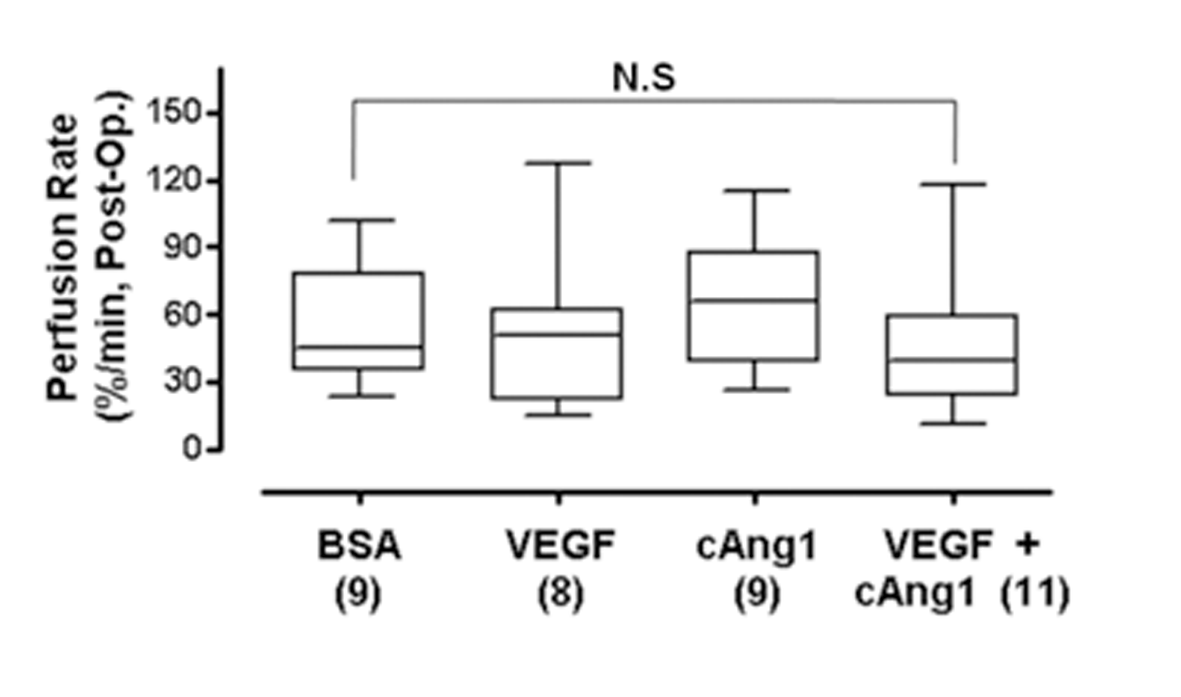

Supplement: Figure S4 — Post-operative perfusion rates in the ischemic limbs of four groups of mice that were distributed evenly for therapeutic angiogenesis study. Note the non-significant difference in perfusion rates at post-op among the groups [ANOVA, F(3,33) = 0.667, p = 0.578, Bonferroni Post hoc: p = 1 among all groups]. (0.16 MB TIF) [file pone.0004275.s005.tif]

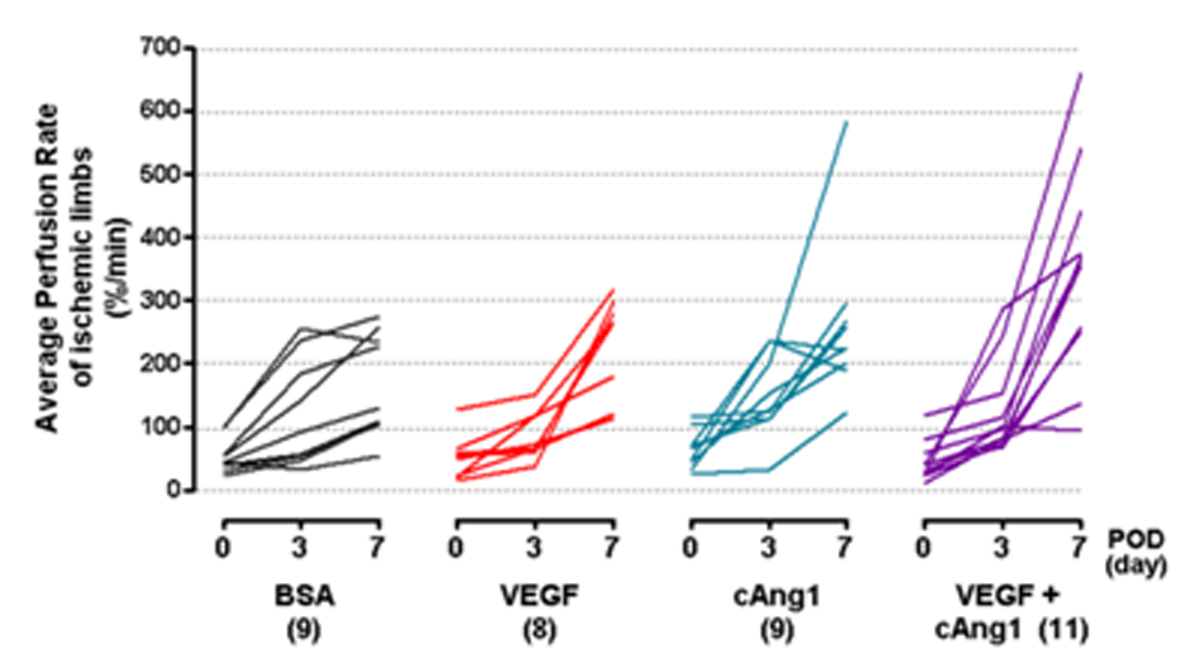

Supplement: Figure S5 — Each line indicates the time-dependent change of the average perfusion rate of ischemic hindlimbs from each mouse. (0.33 MB TIF) [file pone.0004275.s006.tif]
